# Bioprospecting *Kluyveromyces marxianus* as a Robust Host for Industrial Biotechnology

**DOI:** 10.3389/fbioe.2022.851768

**Published:** 2022-04-20

**Authors:** Muhammad Bilal, Liyun Ji, Yirong Xu, Shuo Xu, Yuping Lin, Hafiz M. N. Iqbal, Hairong Cheng

**Affiliations:** ^1^ School of Life Science and Food Engineering, Huaiyin Institute of Technology, Huaian, China; ^2^ State Key Laboratory of Microbial Metabolism, School of Life Sciences and Biotechnology, Shanghai Jiao Tong University, Shanghai, China; ^3^ National Center of Technology Innovation for Synthetic Biology, Tianjin, China; ^4^ Tecnologico de Monterrey, School of Engineering and Sciences, Monterrey, Mexico

**Keywords:** *Kluyveromyces marxianus*, genome editing, food, enzymes, bioethanol

## Abstract

*Kluyveromyces marxianus* is an emerging non-conventional food-grade yeast that is generally isolated from diverse habitats, like kefir grain, fermented dairy products, sugar industry sewage, plants, and sisal leaves. A unique set of beneficial traits, such as fastest growth, thermotolerance, and broad substrate spectrum (i.e., hemi-cellulose hydrolysates, xylose, l-arabinose, d-mannose, galactose, maltose, sugar syrup molasses, cellobiose, and dairy industry) makes this yeast a particularly attractive host for applications in a variety of food and biotechnology industries. In contrast to *Saccharomyces cerevisiae*, most of the *K. marxianus* strains are apparently Crabtree-negative or having aerobic-respiring characteristics, and unlikely to endure aerobic alcoholic fermentation. This is a desirable phenotype for the large-scale biosynthesis of products associated with biomass formation because the formation of ethanol as an undesirable byproduct can be evaded under aerobic conditions. Herein, we discuss the current insight into the potential applications of *K. marxianus* as a robust yeast cell factory to produce various industrially pertinent enzymes, bioethanol, cell proteins, probiotic, fructose, and fructo-oligosaccharides, and vaccines, with excellent natural features. Moreover, the biotechnological improvement and development of new biotechnological tools, particularly CRISPR–Cas9-assisted precise genome editing in *K. marxianus* are delineated. Lastly, the ongoing challenges, concluding remarks, and future prospects for expanding the scope of *K. marxianus* utilization in modern biotechnology, food, feed, and pharmaceutical industries are also thoroughly vetted. In conclusion, it is critical to apprehend knowledge gaps around genes, metabolic pathways, key enzymes, and regulation for gaining a complete insight into the mechanism for producing relevant metabolites by *K. marxianus*.

## 1 Introduction

Yeasts are a heterogeneous group of eukaryotic fungi that have diverse applications in biotechnological, food, environmental, and pharmaceutical fields since the ancient times (Bilal et al., 2020; Mamaev and Zvyagilskaya, 2021). Yeasts are endowed with a set of unique advantageous attributes, such as high production, growth on a variety of feedstocks, ability to compartmentalize reactions in organelles, potential to execute many posttranslational modifications, easy cultivation in small vessels or large-scale bioreactors, facile product separation, and resistance to infectious agents (i.e., bacteriophages) (Cheng et al., 2018; Wang et al., 2020; Staniszewski and Kordowska-Wiater, 2021). All these traits render yeasts, particularly attractive candidates for broad-spectrum applications in various industries (Wagner and Alper, 2016). Although many yeast species have been isolated and identified, the industrial or biotechnological applications are restricted to only a few species, primarily belonging to *Pichia pastoris*, *Saccharomyces cerevisiae*, *Candida utilis*, *Yarrowia lipolytica*, and *Kluyveromyces marxianus* and its asexual forms (e.g., *K. bulgaricus*, *K. fragilis*, *C. kefyr*, and *C. pseudotropicalis*) (Lane and Morrissey, 2010; Wagner and Alper, 2016; Chi et al., 2019). Among these, *S. cerevisiae* holds a prominent place and a largely exploited microbial platform in the biotechnological field owing to its well-annotated genome, availability, well-known physiological traits, ease of use, and genetic manipulability (Lyu et al., 2021). In addition to this traditional yeast, various other non-conventional yeasts with advantageous features for industrial bioprocesses including *P. pastoris*, *K. lactis*, *H. polymorpha*, and *Y. lipolytica* have also gained importance (Gellissen et al., 2005; Wagner and Alper, 2016). Among these non-conventional strains, the *Kluyveromyces* genus has recently emerged as a representative candidate for many applications in food, environment, and biotechnology Duan et al., 2019; Zhou et al., 2018; Hua et al., 2019).

In contrast to *S. cerevisiae*, most of the *K. marxianus* strains are Crabtree-negative or having aerobic-respiring characteristics, and unlikely to endure aerobic alcoholic fermentation. This could be an advantageous characteristic for the large-scale biosynthesis of compounds whose products are associated with biomass formation because the formation of ethanol as an undesirable byproduct can be evaded under aerobic conditions (Wagner and Alper, 2016). Moreover, *K. marxianus* can metabolize a broad spectrum of low-cost feedstocks, such as cheese whey or dairy industry wastes owing to its exceptional physiological features and aptitude to produce heterologous proteins. This thermotolerant yeast is capable of growing at elevated temperatures, with a fast growth efficiency of 0.99/h at 40°C (Banat et al., 1992). The fermentation process at high temperatures can markedly decrease the cooling cost and contamination risk. Simultaneous saccharification and fermentation of lignocellulosic and other polysaccharide-based feedstocks at higher temperature can augment the enzyme efficacy and reducing the enzymes-associated costs. All these desired traits, such as fastest growth, thermotolerance, and broad substrate spectrum (i.e., hemi-cellulose hydrolysates, xylose, l-arabinose, d-mannose, galactose, maltose, sugar syrup molasses, cellobiose, dairy industry waste, such as cheese whey, lactose, and galactose) constitutes *K. marxianus*, a versatile host for applications in the food, feed, and pharmaceutical industries (Löser et al., 2013; Morrissey et al., 2015; Mo et al., 2019a; Mo et al., 2019b; Lyu et al., 2021).

**FIGURE 1:**
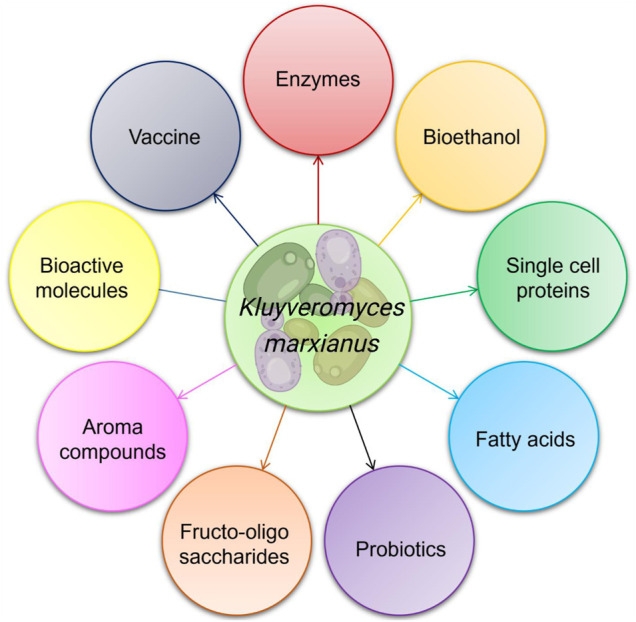
Array of value-added functional bioproducts obtained from *Kluyveromyces marxianus*.

Increasing worldwide demands for biomolecules to be used in foods and other products necessitate exploiting the *K. marxianus* potential in biotechnological applications, including enzymes production, ethanol fermentation, single-cell protein, vaccine preparation, and probiotics (Figure 1). However, the lack of fundamental information about its genetics and physiology is the major drawback for its advancement (Morrissey et al., 2015). This scenario is quickly changing, where significant progress is being devoted to optimizing cultivation conditions and fermentation processes. Moreover, evolutionary or rational engineering approaches have also been applied to develop new strains with unique attributes.

Studies regarding the yeast, *K. marxianus*, are increasing in the recent years. Based on transcriptome analysis, Diniz et al. (2017) analyzed the metabolic flow through the central metabolic pathways and found impaired under the ethanol stress. They observed that the genes involved in ribosome biogenesis are downregulated and gene-encoding heat shock proteins are upregulated. These results can provide evidence to restructure *K. marxianus* to obtain higher ethanol tolerance of *K. marxianus* than S. cerevisiae, which makes *K. marxinus* a more robust host producing ethanol than S. cerevisiae. To widen the substrate range, Anandharaj et al. (2020) constructed *K. marxianus* to express the largest cellulosome complex on the cell surface of *K. marxianus*, where 63 enzymes were displayed on the surface, enabling this yeast to efficiently degrade cellulosic substrates and accumulate 3.09 and 8.61 g/L of ethanol from avicel and phosphoric acid-swollen cellulose, respectively. Recently, *K. marxianus* was rationally engineered to produce aromatic products, such as 2-phenylethanol (2-PE), by a series of metabolic engineering strategies, including modification and overexpression of the pathway to be resistant to feedback inhibition, for overproducing phenylalanine *de novo* from synthetic minimal medium (Rajkumar and Morrissey, 2020). Gene resources and transcriptome data of *K. marxianus* are particularly useful for fundamental and applied research for innovative applications, bearing beneficial properties of temperature resistance, wide-range bioconversion ability, and production of recombinant proteins (Lertwattanasakul et al., 2015). In this review, we provide the current insight into the potential applications of *K. marxianus* for producing an array of various industrially pertinent bioproducts. The CRISPR–Cas9-assisted precise genome editing in *K. marxianus* is also delineated for broadening the scope of *K. marxianus* in biotechnology.

## 2 Applications in Biotechnological Products

### 2.1 Enzyme Production

**FIGURE 2:**
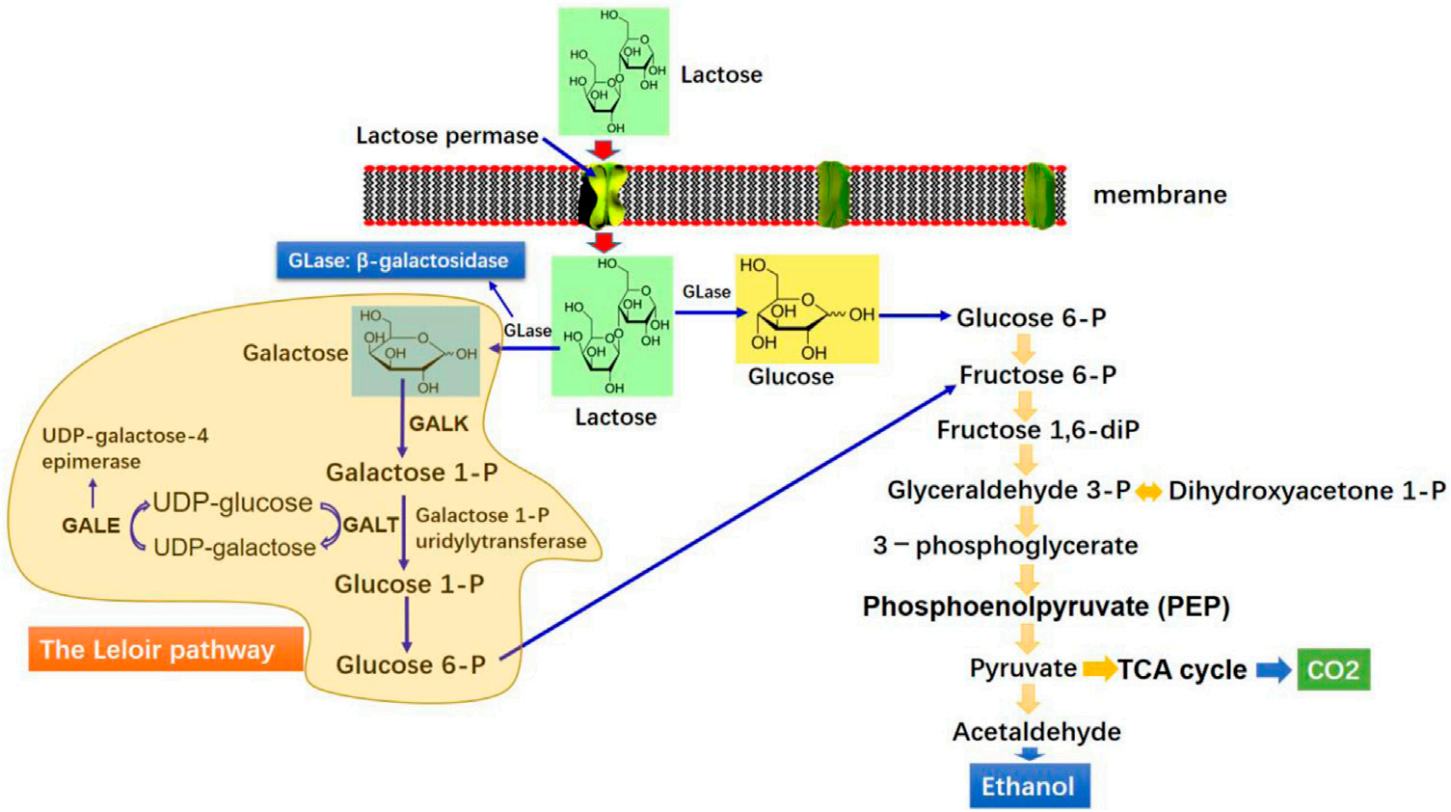
Schematic illustration of the catabolic pathway of lactose and ethanol production by *K. marxianus*.

*Kluyveromyces marxianus* is regarded as an emerging host platform for synthesizing extracellular proteins owing to its capability to grow on an array of cheap feedstocks, including whey, molasses, and spent sulphite liquor (Fonseca et al., 2008). It is also capable of growing on various polysaccharides, such as inulin and pectin (Hensing et al., 1994). *K. marxianus* exhibits inherent ability to produce enzymes because all complex materials stated earlier can be extracellularly hydrolyzed to monomers (Chi et al., 2011). The *K. marxianus* growth on inulin and sucrose occurs *via* the catalytic action of extracellular enzymes, mainly inulinase (Rouwenhorst et al., 1988). The degradation of lactose by β-galactosidases excretion is promising because of simultaneous synthesis of biologically active ingredients, biomass, and enzyme biocatalysts of industrial importance (Fonseca et al., 2008; Chi et al., 2011). A recent summary of different enzyme productions by *K. marxianus* strains is depicted in Table 1. Figure 2 shows the schematic illustrations of uptake mechanism and metabolism of lactose in *K. marxianus*.

**TABLE 1:** Summary of different enzyme productions by *Kluyveromyces marxianus* strains.

Strain name	Name of enzyme	Engineering technique	Cultivation conditions	Enzyme activity	References
*Kluyveromyces marxianus* CCT 3172	β-Galactosidase	-	Temperature 30°C, lactose utilized by yeast, 15.4%	1.10 U/mg	Alves et al. (2019)
			Cheese whey as a substrate		
*Kluyveromyces marxianus* ATCC 16045	β-Galactosidase	-	Culture medium containing whey and parboiled rice effluent was formulated for maximizing the β-galactosidase. production. Cultivation conditions were 10% inoculum, temperature 30°C, and 180 rpm for 72 h	10.4 U/ml	Braga et al. (2012)
*Kluyveromyces marxianus* IFO 0288	Lipase	-	Fermentation time 65 h, optimal nutritional (0.5% olive oil), and cultivation (pH 6.5, 35°C) conditions using the conventional optimization approach		Stergiou et al. (2012)
*Kluyveromyces marxianus* NRRL-Y-7571	Pectinases	-	Liquid medium YNB-pectin (0.5% yeast extract, 1% citric pectin, 0.67% yeast nitrogen base, and pH 5.0)		Piemolini-Barreto et al. (2015)
			Duran flasks (250 ml) were inoculated with *K. marxianus* NRRL-Y-7571 and incubated at 28°C for 48 h, under static conditions		
*Kluyveromyces marxianus*	β-Glucosidase	-	Fermentation temperature 35°C, cultivation time 98 h, inoculum level 10%, and 30 g/L of okara	4.5 U/mg	Su et al. (2021)
*K. marxianus* strain FIM-1∆U	Endo-1,4-β-glucanase RuCelA	Promoter and signal sequence engineering	Fed-batch fermentations in a 5-L bioreactor, temperature 30°, pH 5.5, and dissolved oxygen concentration above 10% of air saturation	24 U/ml	Zhou et al. (2018)
		T(-361)A mutation inside the promoter			
		Deletion of AT-rich region inside 5′UTR (UTR∆A), P10L substitution in the signal sequence			
*K. marxianus* strain FIM-1∆U	Endo-1,4-β-endoxylanase Xyn-CDBFV	Promoter and signal sequence engineering	Fed-batch fermentations in a 5 L bioreactor, temperature 30°, pH 5.5, and dissolved oxygen concentration above 10% of air saturation	25,600 U/ml	Zhou et al. (2018)
		T(-361)A mutation inside the promoter			
		Deletion of AT-rich region inside 5′UTR (UTR∆A), P10L substitution in the signal sequence			
*K. marxianus* strain FIM-1∆U	Endo-1,4-β-mannanase MAN330	Promoter and signal sequence engineering	Fed-batch fermentations in a 5 L bioreactor, temperature 30°, pH 5.5, and dissolved oxygen concentration above 10% of air saturation	10,200 U/ml	Zhou et al. (2018)
		T(-361)A mutation inside the promoter			
		Deletion of AT-rich region inside 5′UTR (UTR∆A), P10L substitution in signal sequence			
Kluyveromyces marxianus NRRL Y-8281	Tannase	-	For SSF, 1 ml of inoculum (10^8^ cells/ml) was inoculated in 250-ml Erlenmeyer flasks containing 5 g of sterilized olive pomace waste, and incubated for 48 h at 45°C	12,711.00 U/ml	Mahmoud et al. (2018)
*Bacillus subtilis* NRRL B-4219 and *K. marxianus* NRRL Y-8287	Xylanase	-	Fermentation medium (50 ml per 250-ml flasks) with 24-h grown cultures of 4% *B. subtilis*, 4% *K. marxianus* were incubated at 35°C and 130 rpm for 24 h by submerged fermentation. pH 7.0	49.5 IU/ml	Yardimci and Cekmecelioglu (2018)
			40% solid load of hazelnut shells		

#### 2.1.1 Inulinases

Inulinase (EC 3.2.1.7) is extensively employed to hydrolyze inulin for producing fructose, fructo-oligosaccharides, and bioethanol, which are the imperative ingredients in pharmaceutical and food industries. Various microbial strains, such as yeast: *K. marxianus* (Selvakumar and Pandey, 1999), filamentous fungi: *Aspergillus fumigatus*, *A. niger*, *Rhizopus* sp., *Penicillium* sp. (Ohta et al., 2002; Moriyama and Ohta, 2007; Kango, 2008), and bacteria: *Staphylococcus sp.*, *Bacillus* sp., *Streptomyces* sp., *Pseudomonas*, and *Xanthomonas* (Selvakumar and Pandey, 1999; Kalil et al., 2005; Gao et al., 2009) have been used to produce inulinases. Nonetheless, *K. marxianus* has been considered the most prodigious host to produce a commercial scale inulinase among all the inulinase-producing microbial strains (Kalil et al., 2005; Zhang et al., 2012).

Recently, Hoshida et al. (2018) investigated the biosynthesis of extracellular proteins by *K. marxianus* using culture media comprising various sugars, such as xylose, glucose, and galactose. Inactivation of INU1 in DMKU3–1042 resulted in the disappearance of extracellular protein and growth retardation in inulin and sucrose media, representing the extracellular protein as an inulinase (sucrase). Among the 16 *K. marxianus* strains analyzed, six strains produced a high level of inulinase in xylose than glucose-based fermentation medium. Nevertheless, a lower activity of INU1 promoter in the xylose medium than glucose medium suggests the controlling of elevated inulinase production in a posttranscriptional manner. Cultures with more agitation also led to produce high titer of inulinase, indicating that oxygen supplementation might influence the inulinase production. Overall, both xylose and oxygen supply improve the titer of extracellular inulinase. An inulinase gene (*INU1* gene) was overexpressed in *K. marxianus* to attain hyperproduction of inulinase and ultrahigh-fructose syrup. Overexpression was accomplished by codon optimization following assortment of an appropriate promoter, and inulinase enzyme was synthesized by a high-cell density fermentation mode. Results revealed that codon optimization of the native *INU1* gene generated an engineered strain KM-N70 (harboring the optimized *INU1N* gene) that was able to synthesize a high titer of the inulinase (251.4 U/ml). The inulinase activity reached 338.5 U/ml by a recombinant KM-KN16 strain carrying the optimized *INU1N* gene with a native *TPS1* promoter from *K. marxianus* KM-0. KM-KN16 strain produced 374.3 U/ml of inulinase within 24 h in 10-L fermentation, while the inulinase titer reached 896.1 U/ml by the recombinant KM-KN16 strain during high-cell density fed-batch fermentation. Furthermore, over 90% of inulin was degraded to glucose and fructose monomers in tubers of Jerusalem artichoke extract by the catalytic action of inulinase preparation in 8 h. Together, the outcomes render recombinant KM-KN16 as the highest producer of inulinase than other bacterial, fungal, and yeast strains (Zhang et al., 2019).

By increasing the glucose concentration from 10 to 20 g/L, the activity of inulinase by *K. marxianus* KM-0 was greatly enhanced; however, further glucose increments to 80 m/L decreased the inulinase activity that might be ascribed to the repressive effect of glucose on the biosynthesis of enzyme. Transcriptional repressor Mig1 encoded by MIG1 is likely to play a major role for glucose repression. Nevertheless, recombinant *K. marxianus* KM-69 (inactivated MIG1 gene) yielded a higher inulinase titer (101.7 U/ml) than non-modified *K. marxianus* KM-0 strain (84.3 U/ml). Overexpressing native inulinase gene from KM-0 into KM-69 additionally improved the inulinase titer to 119.3 U/ml (Zhou et al., 2014). For further enhancing the production of inulinase by the yeast, derepression of glucose repression, and overexpression of the native inulinase gene are important. After disruption of the MIG1 gene in the *K. marxianus* KM-0, the resultant glucose-derepressed mutant (KM-69) yielded 94.6 U/ml of inulinase in the inulin-based fermentation medium within 36 h. Afterward, the overexpression of a native inulinase gene in the KM-69 mutant further increased the level of inulinase. During the 10-L fermentation, the engineered KM-526 strain produced 133.5 U/ml within a short period of 24 h. Inulinase secreted by recombinant KM-526 effectively catalyzed the transformation of inulin into monosaccharides compared to *K. marxianus* KM-0-derived counterpart. Therefore, disruption of the MIG1 gene together with native INU1 gene overexpression is meaningful for enhancing the yield of inulinase in *K. marxianus* KM-0 in short duration, indicating its usefulness in food industries (Zhou et al., 2014).

#### 2.1.2 β-Galactosidases

β-Galactosidases (EC 3.2.1.23) are among the most widely utilized biocatalysts in the food industry. These are also referred to as lactase involved in hydrolyzing lactose to a mixture of glucose and galactose. These enzymes have enormous applications in the food and pharmaceutical industries and can be applied for whey saccharification and milk treatment to reduce lactose (Singh et al., 2016). Lactose reduction is primarily useful for the individuals with inherited disorders for lactose breakdown, such as the black people in Central Africa, Brazil, and United States (Bayless et al., 2017). A plethora of *Kluyveromyces* strains have been described as large-scale producers of β-galactosidases. A number of different approaches with diverse cultivation strategies have been attempted for the biosynthesis of β-galactosidases from cheese whey and molasses (Morrissey et al., 2015; Padilla et al., 2015). Indispensable food processing-related activities like hydrolytic and transgalactosylic activities are generally executed by the use of commercial β-galactosidases (Pivarnik et al., 1995). Enzyme-mediated hydrolysis is implicated for lactose reduction in milk in the food sector, whereas the transgalactosylation is carried out for synthesizing galactose and galacto-oligosaccharides-containing products (Oliveira et al., 2011). Padilla et al. (2015) reported the successful lactulose oligosaccharides production *via* transgalactosylated whey permeate isomerization using the β-galactosidases from *K. marxianus* and *K. lactis*.

The expression of β-galactosidases in *K. marxianus* is regulated by its natural inducers, lactose, and galactose (Liu et al., 2021). Nonetheless, β-galactosidases production depends on the concentration of substrate. The repressive mechanism was superimposed to the substrate inducing effects when the strain was exposed to an elevated level of d-glucose, or lactose, resulting in reduced β-galactosidases activity. This may be attributed to the accumulation of glycolysis metabolite intermediate when microbial cells assimilate d-glucose or lactose with a high rate (Martins et al., 2002). Zhou et al. (2013) observed the suppressed biosynthesis of β-galactosidase in the presence of glucose; however, the production level was restored by the elimination of the MIG1 gene. After disruption of the MIG1 gene, the resulting *K. marxianus* KM-15 strain accumulated a high titer of β-galactosidase compared to the native strain. Under the optimized processing conditions, galactosidase activity by the disruptant KM-15 reached 121 U/ml within 60 h. The fermentative potential of okara (soybean residue) feedstock was explored to produce β-glucosidase by *K. marxianus* (Su et al., 2021). It was found that okara notably induced the synthesis of β-glucosidase reaching 4.5 U/mg under the optimal fermentation conditions of okara 30 g/L, temperature 35°C, inoculum size 10%, and cultivation duration 98 h. The enzyme was a dimer of 66 kDa with two different subunits (44 and 22 kDa). It presented high-acid–alkali resistance, thermal stability, and inhibition tolerance to DMSO (10–50%) (Su et al., 2021).

#### 2.1.3 Lipases

Lipases or triacylglycerol acyl hydrolases (EC 3.1.1.3) are a fascinating class of hydrolytic enzymes, which mediate the hydrolysis as well as synthesis of esters. Generally, they catalyze the breakdown of acyl glycerides that are prerequisite for lipids (triacylglycerol) bioconversion (Vakhlu, 2006). Lipases are endowed with a set of unique attributes including regiospecificity, stereospecificity, substrate specificity, and capability of performing reactions at the interface of water soluble and insoluble systems (Sharma et al., 2002). Similar to proteases or carbohydrases, microbial originated lipases are of prodigious industrial importance owing to high stability than animal or plant counterparts and bulk production at low cost (Vakhlu, 2006). Moreover, lipases from microbial sources offer broad spectrum biocatalytic activities, rapid microbial growth on cheap media, easy genetic amendments, and lack of seasonal fluctuations (Šiekštelė et al., 2015). Among the bacterial and fungal strains, fungal lipases are widely explored, thanks to their unique features, such as efficient extraction procedure, substrate specificity, thermal and pH tolerance, and activity preservation in organic media (Sarmah et al., 2018).

Although the production of lipase is predominant among yeasts, only a few species are able to secrete lipases with desired characteristics and inadequate amount for industrial exploitation. *Y. lipolytica*, *Candida rugosa*, *Candida utilis*, *Candida antarctica*, *Saccharomyces*, and *K. marxianus* have been found as the yeasts with high-lipase synthesizing capabilities (Sarmah et al., 2018). Deive et al., 2003) assessed the capability of *K. marxianus* for the production of lipases and evaluated the influence of surfactants and various lipidic compounds on the synthesis and secretion of enzyme. A complex liquid medium containing different inducers, like fatty acids and triacylglycerols supported the maximum production of lipases by *K. marxianus*. The utmost enzyme titer (80 U/ml in 3 days) was recorded in the medium with the inclusion of 5 g tributyrin/L and 2 g urea/L. However, the inclusion of surfactants does not have favorable effect on the production.

In addition to physicochemical parameters like temperature, pH, and dissolved oxygen, medium composition can also significantly affect the lipase production from *K. marxianus*. For example, Stergiou et al. (2012) evaluated the optimization of various processing factors that influence the production of extracellular lipase by *K. marxianus* IFO 0288. Results revealed that the production was 18-fold increased using optimal cultivation (35°C, pH 6.5, 150 rpm) and nutritional (0.5% olive oil) conditions after the 65-h fermentation of olive oil as a growth substrate. In another study, *K. marxianus* 83F presented higher lipase production activity in Serro Minas cheese, indicating it as a promising starter culture for cheese production owing to desired sensory features (Cardoso et al., 2015). Due to the presence of high fatty acids contents, avocado oil was tested as an inducing agent to synthesize lipolytic enzymes from *K. marxianus* L-2029. For determining the best avocado oil levels in the yeast medium, induction was performed for 24 h using different concentrations. *K. marxianus* L-2029 accumulated the highest productivity of extracellular lipase at 3.5% v/v avocado oil, pH 6, and 36°C (Martinez-Corona et al., 2019). In another study, the same authors carried out a bioinformatics analysis of genes from *K. marxianus* L-2029 for the identification and characterization of extracellular lipases. By *in silico* translation, amino acid sequence of 10 putative lipases was obtained ranging between 389 and 773 amino acids. Among these, two putative proteins exhibited a signal peptide, 33 and 25 amino acids long for *KmLIP3p* and *KmYJR107Wp*, and a molecular mass of 58.23 and 44.53 kDa, respectively. From the 3D models of *K. marxianus* putative L-2029 lipases, the conserved pentapeptide was determined to be GHSLG for *KmYJR107Wp and* GTSMG for *KmLIP3p*. The phylogenetic analysis of all *K. marxianus* lipases demonstrated evolutionary affinities with lipases from abH23.01, abH23.02, and abH15.03 and families (Martínez-Corona et al., 2020). In fact, microbial lipases are an interesting class of biocatalysts with prospective applications in medical and pharmaceutical, detergent, food, baking, dairy, flavor, organic synthesis, paper manufacturing, perfumery, cosmetics, leather, biosurfactant, agrochemical, oleochemical, bioremediation, and biosensors related industries (Sarmah et al., 2018).

#### 2.1.4 Pectinases

Endo-polygalacturonases (EC 3.2.1.15), generally referred to as pectinases, catalyzes the hydrolysis of heteropolysaccharide pectins that comprise the major structural component of the plant cell walls. Due to cell wall degradation abilities, pectinases find increasing applications in the wine and juice manufacturing (Alimardani-Theuil et al., 2011). Among the pectinase extracted from plants and various microbial strains including filamentous fungi, yeasts, and bacteria, yeast pectinases have gained considerable importance in the recent past. Pectinolytic yeasts can synthesize diverse types of pectinolytic enzymes depending on the genetic background and environmental conditions. Four yeast species, *S. cerevisiae*, *S. fragilis*, *Torulopsis kefir*, and *K. marxianus* have been largely studied to produce pectinolytic enzymes. These enzymes include pectin lyases (PL), polygalacturonase (PG), pectate lyase (PaL), and pectinesterase (PE) based on the pH, temperature, substrate availabilities. For example, *Kluyveromyces, Saccharomyces*, and *Candida* synthesize PG (i.e., endopolygalacturonase), while PG and PE are the major enzymes secreted by *Rhodotorula* (Alimardani-Theuil et al., 2011). Oliveira et al. (2012) investigated the impact of various pectic feedstocks, temperature, pH, and glucose on the polygalacturonase biosynthesis by *K. marxianus* CCMB 322. The enzyme secreted in the culture filtrate of *K. marxianus* CCMB 322 was found to be partially constitutive, exhibiting optimal temperature and pH of 70°C and 7.36, respectively. It retained over 90% of its initial activity for 50 min at a high temperature of 50°C. It was found that supplementation of glucose in the medium can affect the regulation of polygalacturonase synthesis. In order to determine optimal pH, temperature, and incubation time for pectinase production by *K. wickerhamii*, Moyo et al. (2003) applied a central composite rotatable design. pH and temperature were found to be the most influential factors that affect the enzyme synthesis. The best enzyme yield was recorded at pH 5.0 and 32°C after fermentation duration of 91 h. The pectinolytic enzyme produced was referred to as polygalacturonase (PG), thermotolerant at different temperatures. Its activity was enhanced by Ca^2+^ ions; however, Zn^2+^, Mg^2+^, Na^+^, Mn^2+^, and Co^2+^ ions inhibited the activity (Moyo et al., 2003).

### 2.2 Bioethanol Production

Fermentative production of bioethanol at elevated temperatures has gained attention because the rapid fermentation rate at high temperature is likely to minimize the cooling cost, circumvent the contamination risk, and enables simultaneous saccharification and fermentation (Nonklang et al., 2008). The optimal *K. marxianus* growth at a high temperature is particularly alluring to facilitate cooling during the extensive level fermentations for which transferring heat is a restrictive factor. Current industrial production of ethanol mainly employs *S. cerevisiae* due to its superior production titer and tolerability to high ethanol concentrations (about 120 g/L). Nevertheless, the optimal temperature of this yeast is relatively low (25–30°C) (Qiu and Jiang, 2017). Therefore, much interest has recently been geared to explore yeast species that are capable of producing ethanol at higher temperatures, and *K. marxianus* appears a robust isolate for this purpose (Figure 2) (Madeira-Jr and Gombert, 2018). Table 2 gives a recent summary of ethanol production by various *K. marxianus* strains. *K. marxianus* species are thermotolerant and can grow at 47 (Nonklang et al., 2008; da Silva et al., 2018), 49 (Hughes et al., 1984), and even 52°C (Banat et al., 1992). It is able to produce ethanol at temperature above 40°C (Madeira-Jr and Gombert, 2018), and assimilate a wide range of cheap substrate, including molasses, whey permeates (Hang et al., 2003; Ozmihci and Kargi, 2007; Martínez et al., 2017), and corn silage juice to synthesize ethanol. Given all these features, *K. marxianus* is appreciated as a prospective alternative to *S. cerevisiae* for commercial scale ethanol production.

**TABLE 2:** Summary of ethanol production by various *Kluyveromyces marxianus* strains.

Strain name	Substrate	Pretreatment technique	Fermentation condition	Ethanol titer (g/L)	References
*Kluyveromices marxianus* (Kmx24)	Sweet whey	-	4.50 g/L of precursor, 0.76 g/L of salt, and fermentation duration of 48 h	1.2 (2-phenylethanol)	Alonso-Vargas et al. (2022)
*Kluyveromices marxianus* JKSG-6	Cotton stalk	Sequential dilute acid–alkali pretreatment using sulfuric acid (1%, v/v) and sodium hydroxide (3%, w/v)	For SSF, flasks were incubated at 42°C. PSSF experiments were conducted by performing a 12-h enzymatic pre-saccharification at 50°C, following temperature reduction to 42°C before yeast inoculation	26.10	Kaushik et al. (2022)
*Kluyveromices marxianus* MM III-41	Green coconut shell	Hydrothermal	Fermentation was carried out at 37°C for 24 h and 75 rpm	8.83	Gomes et al. (2021)
			Using 100 ml of reaction medium		
*Kluyveromices marxianus* MM III-41	Green coconut shell	Acidic	Fermentation was carried out at 37°C for 24 h and 75 rpm	9.71	Gomes et al. (2021)
			Using 100 ml of reaction medium		
*Kluyveromices marxianus* MM III-41	Coconut-tree leaflet	Hydrothermal	Fermentation was carried out at 37°C for 24 h and 75 rpm	10.26	Gomes et al. (2021)
			Using 100 ml of reaction medium		
*Kluyveromices marxianus* MM III-41	Coconut-tree leaflet	Acidic	Fermentation was carried out at 37°C for 24 h and 75 rpm	7.01	Gomes et al. (2021)
			Using 100 ml of reaction medium		
*Kluyveromices marxianus* MM III-41	Coconut-tree leaf stalk	Hydrothermal	Fermentation was carried out at 37°C for 24 h and 75 rpm	12.99	Gomes et al. (2021)
			Using 100 ml of reaction medium		
*Kluyveromices marxianus* MM III-41	Coconut-tree leaf stalk	Acidic	Fermentation was carried out at 37°C for 24 h and 75 rpm	7.44	Gomes et al. (2021)
			Using 100 ml of reaction medium		
*Kluyveromyces marxianus* DMKU-KS07	Cassava chip hydrolysates and molasses	-	Modified simultaneous saccharification and fermentation with co-fermentation of substrates from the enzymatic hydrolysates of raw cassava chips at 42°C for 12 h	118	Lomthong et al. (2021)
*K. marxianus* ETP87	Shola dairy	-	Whey fermentation was conducted in a 250-ml flask containing 100 ml crude whey	12.49	Tesfaw et al. (2021)
			The media in the flasks were inoculated with *K. marxianus* ETP87 seed cultures and allowed to grow aerobically		
			Fermentation was carried at 30°C on a water bath shaker at 100 rpm		
*Kluyveromyces marxianus*	Azolla weed hydrolysate	Thermal acid hydrolysis	Ethanol fermentation was conducted with Azolla weed hydrolysate (100 ml) in a 250-ml Erlenmeyer flask under semianerobic conditions	26.8	Chupaza et al. (2021)
			Azolla weed hydrolysates were fermented at 30°C and 150 rpm		
*Kluyveromyces marxianus*	Banana peels	Pretreatment at 121°C for 15 min	Simultaneous saccharification and fermentation (SSF) were executed in 100-ml Erlenmeyer flasks using 10% (w/v) of autoclaved banana peels with 2 g/L yeast at pH 4.8	11	Palacios et al. (2021)
			Assay was performed for 24 h in a rotatory shaker at 150 rpm at 41°C		
*Kluyveromyces marxianus* MTCC 4139	Cassava peel	Alkali-assisted hydrothermal pretreatment	Fermentation media was autoclaved at 110°C for 15 min and 10% (v/v) *K. marxianus* inoculum was aseptically added to the fermentation medium. The separate hydrolysis and fermentation were carried out at 30 for 72 h at 150 rpm	(0.44 g/g)	Papathoti et al. (2021)
*Kluyveromyces marxianus* MTCC 4139	Cassava peel	Alkali-assisted hydrothermal pretreatment	Simultaneous saccharification and fermentation was initiated by aseptically adding 10% (v/v) *K. marxianus* inoculum, following enzymatic solution containing cellulase (10 FPU/g substrate), α-amylase, and glucoamylase (each containing, 450 IU/g substrate)	(0.41 g/g)	Papathoti et al. (2021)
			SSF was performed at 100 rpm, 40°C for 72 h		

Production of ethanol from non-food substrate is favorably suggested for avoiding criticism with food supply. Nutrients enriched whey can be considered a non-food low-cost substrate for producing ethanol by *Kluyveromyces* spp. Tesfaw et al. (2021) achieved the optimized ethanol production from crude whey using a newly isolated *K. marxianus* ETP87 strain. The non-sterilized and sterilized whey were utilized as feedstocks to assess the *K. marxianus* ETP87 competition with lactic acid and other microflora. The use of sterilized whey resulted in a slightly higher amount of ethanol than non-sterilized counterpart. Likewise, a substantially higher ethanol titer was achieved from whey without pH adjustment, and yeast accumulated the maximum ethanol between 30 and 35°C. It was capable of producing high level of ethanol until 45°C, and ethanol titer was drastically reduced when temperature was increased at 50°C. Inclusion of ammonium sulfate and peptone stimulated the ethanol production, while yeast extract and urea have negative effect on the ethanol productivity. Based on a circular economy concept, Tinôco et al. (2021) produced cellulosic ethanol by *K. marxianus* CCT 7735. Following acid–base pretreatment and hydrolysis at 50°C for 3 days, the saccharified sweet sorghum was subjected to ethanol production at three different temperatures (37, 42, and 45°C). Precisely, 17.83 g/L ethanol was produced after 24 h at 42°C with a corresponding yield of 2769.8 L/ha_sorghum_, which is double compared to corn straw.

In a recent study, Madeira-Jr and Gombert (2018) demonstrated that the production of bioethanol at elevated temperatures (∼48°C) by *K. marxianus* NCYC 3396 using sugarcane can reduce cooling costs, risk of contamination, use of antibiotics, water usages, consumption of H_2_SO_4_ in cell reprocessing, and the use of energy in distillation, which ultimately reduce the bioethanol production cost in Brazilian biorefinery. Using *K. marxianus*, they achieved a yield of 0.40 g ethanol/g glucose at 48°C that was similar to *S. cerevisiae* CEN.PK113-7D at 37°C. Though the ethanol titer (0.43 g/g glucose) was comparable by *K. marxianus* K213 and *S. cerevisiae* using glucose at 30°C, *S. cerevisiae* lost its ethanol production ability when temperature raised to 45°C, while *K. marxianus* K213 retained the same conversion efficacy (0.43 g/g glucose) at this temperature (Yan et al., 2015). Murari et al. (2019) also corroborated that temperature (32.5–35°C) was the most influential parameter to produce ethanol from cheese whey by *K. marxianus* URM7404 following pH (4.8–5.3) and lactose concentration (61–65 g/L).

Raw starch-degrading enzyme (RSDE) is being employed in the ethanolic fermentation bioprocess for hydrolyzing starchy materials, such as cassava chips and cassava starch (Lomthong et al., 2015; Lomthong et al., 2016). Using RSDE reduces processing costs and energy consumption because the hydrolysis of starch granules is carried out below the gelatinization temperature than traditional starch degradation that occurs at 80–100°C (Cinelli et al., 2015; Lomthong et al., 2018). In a recent study, Lomthong et al. (2016) produced RSDE by *Laceyella sacchari* LP175 and applied to hydrolyze cassava chips at 50°C. The resulting sugar syrup was used as a fermentative substrate for bioethanol production. The saccharification and hydrolysis of cassava chips was carried out at lower temperature without heating-fostered ethanol fermentation by *K. marxianus* DMKU-KS07 at 42°C (Trakarnpaiboon et al., 2017). Similarly, molasses is enriched with high sucrose contents and other nutrients, which are conducive for microbial growth to produce bioethanol. Arshad et al. (2017) utilized molasse substrate for very high gravity (VHG) bioethanol by *S. cerevisiae*, yielding the highest ethanol titer to be 122 g/L. In contrast to other feedstocks, molasses present the benefits of bioethanol production without any pretreatment and saccharification (Chotineeranat et al., 2010). Nevertheless, a high concentration of metal ions impedes the activity of microbial enzymes and might lower ethanol yield (Chotineeranat et al., 2010; Wattanagonniyom et al., 2017). Under non-sterile conditions, Lomthong et al. (2021) evaluated the improvement of VHG bioethanol titer using molasses and cassava chip co-substrate hydrolysates by *K. marxianus* DMKU-KS07 at 42°C. Results showed a high bioethanol concentration of 118 g/L with ethanol yield (YP/S) at 0.44 g EtOH/g total sugar and productivity of 2.19 g/L/h by simultaneous saccharification and fermentation at 42°C for 12 h.

### 2.3 Single-Cell Proteins

Single-cell proteins (SCPs) are referred to as dietary single-cell microorganisms, whose protein extracts or biomass are originated from mixed or pure bacterial cultures, yeasts, mushrooms, or microalgae. These microorganisms can be utilized as food ingredients, dietary supplements, or high protein foods for animal and human consumption (Ritala et al., 2017). Thus, extensive biosynthesis of microbial biomasses could be useful to replace animal- and plant-derived proteins for food or feed owing to high-protein contents, fast proliferation rate, and the aptitude to assimilate a wide spectrum of carbon containing waste materials (Karimi et al., 2018; Karim et al., 2020). Given a shorter microbial life cycle, SCPs can be attained rapidly than that of the proteins produced from agricultural sources. Amid the SCP producers, yeasts have appeared as emerging cell factories due to tiny particle size, large protein content, facile handling, and comparatively minimum production costs. *K. marxianus* is a promising candidate with considerable potential for SCP biosynthesis and thus extensively employed as a feed organism in many countries. Compared to *S. cerevisiae*, *K. marxianus* displays a high specific growth efficiency and highest biomass yield in aerobic continuous fermentation. Yadav et al. (2014a) utilized *K. marxianus* GQ 506972 to produce SCP with a simultaneous COD elimination of cheese whey. They achieved 6 g/L of biomass along with over 50% removal of COD at pH 3.5 and 40°C after 36 h of batch fermentation. Increase in the inoculum level led to increased biomass production of 15 g/L and 80% removal of COD. The same authors also appraised the efficiency of the mixed culture system for obtaining high-quality SCP and enhancing the COD removal in continuous and batch fermentations of whey under cultivation conditions of pH 3.5 and 40°C (Yadav et al., 2014b). The coculture (*C. krusei* and *K. marxianus*) gives rise to 19% greater biomass yield and 33% higher productivity with concurrent 8.8% elimination of COD than the single culture in the batch system. The SCP produced from coculture contained essential amino acids and a desired protein content, indicating a mixed culture prodigious approach for producing SCP and wastewater treatment (Yadav et al., 2014a; 2014b). Yadav et al. (2016) produced food-grade SCPs at pH 6.5 and 30°C using a coculture system of *S. cerevisiae* and *K. marxianus*. In comparison to single culture (84%), the mixed culture resulted in 92% of total whey proteins under the optimal conditions. Many microorganisms including *K. marxianus*, *C. kefyr*, and *S. cerevisiae* have been grown in the food wastes *via* SSF. Among these, *K. marxianus* contains the maximum concentration of fat and protein, and thus might be utilized to enrich livestock feed (Aggelopoulos et al., 2014).

### 2.4 Probiotic Role

Probiotics are the living microorganisms that confer health benefits on the host by administrating inadequate amounts (Hill et al., 2014). These include *Lactobacillus*, *Clostridia*, *Bifidobacterium*, *Enterococcus*, *Faecalibacterium*, and *Propinonibacterium* (Altieri, 2016). Although *S. cerevisiae var. boulardii* has also been considered a probiotic since many years (Moré and Swidsinski, 2015), growing interest is diverted in recent years to explore the probiotic potential of non-conventional yeasts. *K. marxianus* is regarded as a promising probiotic due to its modification capacity in the adhesion, cell immunity, and human gut microbiota. In addition, it also possesses anti-inflammatory, antioxidant, and hypocholesterolemic features (Xie et al., 2015; Cho et al., 2018). It might be able to survive in the digestive tract reaching the intestines to serve as prebiotics due to acid and bile tolerance present in the gastrointestinal environment. *K. marxianus* isolated from kefir presented cholesterol-lowering capability, reducing 30% of cholesterol even greater than *K. lactis* ATCC 34440 and *S. cerevisiae* ATCC 6037 (Cho et al., 2018). *K. marxianus* AS4, obtained from dairy products, that is, cheese and yogurts, has shown excellent resistance to acidic pH, high bile salts, and superior antimicrobial activity. It also revealed outstanding anticancer activity against gastrointestinal tumor cells due to the secretion of biologically active metabolites, downregulating Bcl-2 gene, upregulating a pro-apoptotic protein, and BAD and CASP9 gene expression system (Saber et al., 2017). Among various yeast strains with probiotic efficiency, *K. marxianus* JYC2614 showed the best tolerance to bile salt and acid, as well as good cell surface hydrophobicity and satisfactory auto-aggregation (Hsiung et al., 2021). Recently, Goktas et al. (2021) determined the probiotic properties of yeast species isolated from kefir samples and compared with a commercial probiotic yeast *S. boulardii*. In contrast to *S. boulardii*, Kefir isolates, *K. marxianus* and *S. cerevisiae*, showed hydrophobicity, high auto-aggregation, and antioxidant potentialities. They also demonstrated potent antifungal activities against some mold species. Motey et al. (2020) assessed the probiotic characteristics of *K. marxianus* and *S. cerevisiae*, which were isolated and identified from the West African milk products nunu, lait caillé, and a cereal-based product mawè. Isolates were capable of growing in bile salts with the highest specific growth rate (μ_max_) of 0.58–1.50 hour^−1^. Adhesion of yeast to Caco-2 cells was found to be strain-specific varying between 8.0 and 36.2%. At the gastrointestinal pH environment, *S. cerevisiae* and *K. marxianus* also maintained the pH_i_ homeostasis and increased transepithelial electrical resistance across the Caco-2 monolayers, indicating their appreciable probiotic efficiency in West African fermented products. In another work, *K. marxianus* PTCC = 5189 was utilized for the production of probiotic yoghurt, which was then contaminated with *Penicillium notatum* PTCC = 5074 and *Aspergillus parasiticus* PTCC = 5018. The contaminants and changes in the count of probiotic yeasts during the storage at 4°C at 28 days were assessed and subjected to comparison with the control. Because of *K. marxianus*, the numbers of yoghurt adulterating molds were significantly reduced. *K. marxianus* population was satisfactory after 28 days of storage for providing a probiotic product with 7.35 log CFU/gr (*p* < 0.01), indicating its usefulness to produce probiotic yoghurt on an industrial scale (Oroojzadeh, 2020).

Wang et al. (2017) studied the impacts of *K. marxianus* addition on intestinal structure, immune responses, and growth attributes in broiler chickens. Experimental results revealed that increasing supplementation of *K. marxianus* resulted in reduced feed conversion efficiency but a linear improvement in relative thymus weight, increased serum IgG, and lysozyme levels. Better feed conversion rate and immune function along with intestinal structure in broilers by *K. marxianus* supplementation might be ascribed to the better structure of ileal microbes.

### 2.5 Fructo-Oligosaccharides Production

Fructose is a potential alternate sweetener to sucrose owing to its high sweeting capacity (1.5–2 times than sucrose) and could increase the absorption of iron in children. Fructo-oligosaccharides (FOS) are considered a prospective source of dietary fiber with bifidogenic effects. They are utilized as food ingredients owing to their health beneficial properties such as maintaining a good balance in the intestinal flora and promoting the multiplication of bifidobacterial community in the intestine. Recently, they have granted the “GRAS” status by the FDA (Flores-Gallegos et al., 2015). Both FOS and fructose can be produced through the enzymatic degradation of inulin; however, the synthesis was typically carried out at an elevated temperature (around 60°C) because of inadequate solubility of inulin at room temperature. Therefore, it is of great significance to characterize and isolate thermotolerant inulinases for catalyzing the hydrolysis of inulin at higher temperatures (Flores-Gallegos et al., 2015). Inulin is regarded as a good feedstock for producing inulo-oligosaccharides by endoinulinase-mediated catalytic reactions, but lack of solubility in cold water or slight solubility (only 5%) in water at 55°C remains a major challenge for its hydrolysis. This fact necessitates the exploration of inulinase that are capable of working at higher temperatures for industrial applicability. In this perspective, *K. marxianus* can be considered a robust candidate for FOS production. Struyf et al. (2017) developed a yeast-based approach by an inulinase-producing *K. marxianus* strain that resulted in over 90% fructan reduction in the final product, whereas only 56% fructan was reduced in the case of *S. cerevisiae* strain as a control. In order to develop a high-performing endo-inulinase for FOS biosynthesis from inulin, an inulin-binding module (IBM) was integrated into C- or N- terminal of the enzyme*.* The C-terminal fusion one (Eninu-IBM) with high activity, thermal stability, and inulin binding efficiency was used to catalyze inulin break down at high-temperature in a 10-L bioreactor. Results obtained high purity FOS (91.4%) with FOS titer, productivity, and yield of 717.3 g/L, 358.6 g/L/h and 0.912 gFOS/g inulin, respectively (Mao et al., 2019). Risso et al. (2012) investigated the FOS production by carrier-supported inulinase from *K. marxianus* NRRL Y-7571 in aqueous and aqueous-organic systems using sucrose as a substrate. In the case of an aqueous–organic system, the utmost yield of FOS (18.2 ± S0.9 wt%) was achieved at optimal condition of pH 5.0, 40°C, aqueous/organic ratio (25/75 wt%), enzyme activity (4 U/ml), and sucrose concentration of 60% (w/w), whereas the highest yield of FOS was achieved to be 14.6 ± 0.9 wt% for the aqueous system.

Various yeasts, including *C. kefyr*, *Debaryomyces antarelli*, and *K. fragilis* or *K. marxianus* have been used to produce inulinases (Beluche et al., 1980; Negoro and Kito, 1973; Holyavka et al., 2016). Among these, *K. marxianus* is preferred for fructose biosynthesis because of their growth on fructans, such as inulin; thus, inulinases can hydrolyze plant-originated fructans (Chi et al., 2011). A considerable amount of inulin is present in the tubers of various plants, such as dahlia, yacon, chicory, and Jerusalem artichoke. Biosynthesis of fructose by enzyme-assisted hydrolysis of plant extract-derived oligo- and polysaccharides by *K. marxianus*-immobilized inulinases could be a gainful strategy for large scale production of sugar (Holyavka et al., 2016). Singh et al. (2016) applied duolite A568-immobilized extracellular exoinulinase from *K. marxianus* YS-1 for the production of high-fructose syrup. The catalytic action of biocatalyst generated 39.2 and 40.2 g/L of fructose in 4 h using raw and pure inulins, respectively. In addition, inulinases secreted by *K. marxianus* using xylose medium is another appealing way for producing elevated titer of fructose syrup (Hoshida et al., 2018).

### 2.6 Vaccine Candidates

Porcine circovirus (PCV), belongs to the genus *Circovirus*, family Circoviridae, is among the smallest animal virus consisting of a single-stranded circular DNA (1.7–2 kb). Three genotypes of PCV include PCV1, PCV2, and PCV3. Among these, PCV1 was initially identified and characterized in infected PK15 cells (Tischer et al., 1974). This virus was considered non-pathogenic with widespread infection in all swine-producing areas (Allan et al., 2012). PCV2 is a pervasive kind of virus with considerable pathogenicity and closely related to the postweaning multisystemic wasting syndrome (PMWS), as well as other porcine circovirus diseases (PCVDs), such as respiratory disorder, enteric disease, reproductive failure, porcine dermatitis, and nephropathy syndrome (PDNS), resulting in massive economic losses in the swine industries (Chae 2005; Gillespie et al., 2009; Cruz et al., 2018). PCV3 is a new PCV genotype, which was initially recognized from piglets with cardiac pathology and multisystemic inflammation in the United States (Phan et al., 2016).

Vaccination is regarded as the best strategy for reducing the death rate and improving the cultivation of porcine circovirus disease-affected pig populations (Fachinger et al., 2008). PCV2 virus-like particles (VLPs) are a kind of subunit vaccine, which imitate PCV2 virus morphology and exhibit advantageous features over the inactivated vaccine and live-attenuated vaccines for their safety, non-replicating, and particulate structure (Wu et al., 2012). A number of host platforms, such as insect–baculovirus, *E. coli*, *P. pastoris*, *S. cerevisiae*, and silkworm pupae, have been explored and attempted to express CAP protein for obtaining PCV2 VLPs (Wu et al., 2012; Tu et al., 2013; Nainys et al., 2014; Masuda et al., 2018); however, the cost-effective production remain a major hindrance. Duan et al. (2019) reported PCV2 VLPs preparation by expressing PCV2d CAP protein in *K. marxianus* with a high yield of 1.91 g/L in a 5-L bioreactor. The yield obtained significantly surpassed the PCV2 VLPs achieved by *E. coli*, *P. pastoris*, and baculovirus–insect cell. The PCV2 VLPs was capable of inducing a high titer of anti-PCV2 IgG antibodies in mice serum together with decreasing the virus titers in spleens and livers of the tested mice. Spontaneously assembly of CAP protein into VLPs showed the prospects of cost-effective preparation of PCV2 vaccines. Recently, Yang et al. (2021) used *K. marxianus* to evaluate the expression and downstream processing of PPV VLPs. The VP2 protein from Kresse strain was subjected to expression following optimization by the codon bias of *K. marxianus*. After 48-h fermentation, the PPV VLPs yield reached 2.5 g/L in a 5-L fermenter. Two strategies, including cation exchange chromatography with Sephacryl^®^ S-500 HR chromatography and anion exchange chromatography following cross-flow diafiltration were used for the purification of VLPs from the supernatants. Administration of mice with purified PPV VLPs (95% purity) resulted in the induction of high titers of IgG antibodies in sera, showing hemagglutination inhibitions on both guinea and swine pig erythrocytes. Cytokine’s detection and spleen lymphocyte proliferation suggest *K. marxianus*-induced PPV VLPs triggered the humoral and immune immunity responses in mice.

## 3 Biotechnological Improvement and Developing New Biotechnological Toolsets

Isolation of *K. marxianus* strains from different sources display substantial differences in physiological traits as well as productivity and yield of platform biomaterials and commodity chemicals. Notwithstanding such difference, biotechnology tools, and adaptation to stress, metabolic, or genetic engineering are promising to improve the inherent abilities of *K. marxianus* (Nurcholis et al., 2020). Sharma et al. (2017) carried out evolutionary adaptation on *K. marxianus* NIRE-K3 at 45°C using xylose as a solitary carbon source, yielding a new derivative with considerable enhancement in xylose assimilation, and yields and productivities of xylitol and ethanol. Likewise, an osmotolerant yeast strain with higher lactose assimilation capability from *K. marxianus* MTCC 1389, a strain with high ethanol biosynthesis and fast growth in lactose containing whey, was obtained by adapting to a high level of lactose (Saini et al., 2017). Kim et al. (2019) developed a thermally stable derivative with glucose and xylose co-fermentation capability by the alleviation of catabolite repression through evolutionary approach using 2-deoxyglucose. Increased activity of xylosidase was reported *via* evolutionary adaptation by cultivating *K. marxianus* in a xylose-containing medium (Behera et al., 2016). All these positive outcomes encourage additional in-depth evaluation of evolutionary adaptation approaches. Nevertheless, identification of key mutation points in these mutants remains to be challenging due to the lack of sufficient genomic data for the wild-type strains. Therefore, it is remarkably imperative to acquire comprehensive information on mutation points. A 2-deoxyglucose-tolerant *K. marxianus* DMKU-3-1042-derived mutant has a single-point missense mutation in RAG5 that causes reduced RAG1 expression and increased expression of INU1 (Nguyen et al., 2015; Nurcholis et al., 2019). By ethanol-driven laboratory evolution, Mo et al. (2019) acquired a mutant that possesses high ethanol yield at elevated temperature and ethanol conditions. They proposed a positive relationship between the ethanol production and tolerance by yeast and inferred that improved ethanol resistance is not due to a meaningful mutations or ploidy change but attributed to transcriptional reprogramming based on transcriptome and genome analyses.

Li et al. (2018) tailored *K. marxianus* DMKU3–1042 by introducing one amino acid substitution (K31E) of the TATA-binding protein Spt15. This strategy causes differential expression of many genes related to ethanol tolerance. Transcriptional factors Msn2 and Hsf1 of *K. marxianus* can improve both ethanol fermentation and cell growth of *S. cerevisiae* at higher temperatures (Li et al., 2017). Hua et al. (2019) developed a xylose and glucose co-utilizing strain by integrating gene knockout mutations of SNF1 or HXK1 with overexpressing xylose-specific transporter genes and xylose reductase. Cernak et al. (2018) created a heterothallic *K. marxianus* strain by genetic engineering to carry three important properties of thermo-resistance, lipid biosynthesis, and easy transformation of exogenous DNA. All these traits might result in the development of *K. marxianus* as a robust industrial platform to produce an array of bio-renewable chemicals. In another study, various promoter combinations (PACO1 and PMDH1) and secretion signal peptides (Scα-MFss and KmINUss) were used to construct strains bearing either plasmid-borne or genomically integrated constructs for secretion of a single-chain antibody (scFv) (Nambu-Nishida et al., 2018).

## 4 Mechanistic Insights Into the CRISPR–Cas9 System

The CRISPR–Cas system was first discovered to offer an immunological weapon for archaea and bacteria against invasive bacteriophages (viruses) and mobile genetic elements (Lander 2016; Mojica and Montoliu 2016). Based on effector module organizations, these systems were characterized into two different classes (six types). Among these, the type II CRISPR system from *Streptococcus pyogenes* is being well characterized and studied (Makarova et al., 2017). It is also one of the commonly applied toolboxes for genetic engineering of yeast strains. Cas9 protein is an RNA-assisted endonuclease that catalyze the cleavage of DNA double strands with two active parts— the RuvC and HNH domains. Due to the identification of Cas9 in bacterium, fusion of a nucleus localization sequence (NLS) to Cas9 is required to facilitate targeting the nucleus genomes of eukaryotes (Löbs et al., 2017a). Single-guide RNA (sgRNA) is another necessary constituent that guides Cas9 to targeting sites. The canonical sgRNA contains a trans-activating crRNA (tracrRNA) and a CRISPR-targeting RNA (crRNA). The first 20 base pairs complementary sequence at 5′ end of crRNA is important for Cas9 endonuclease functioning, and three-nucleotide protospacer adjacent motif (PAM). NGG must be found immediately at 3′ end of the desired locus in genome (Murugan et al., 2017). After sgRNA direction, Cas9 targets the genome-specific sequence with PAM, resulting in the cleavage of both DNA strands (Wang et al., 2015). Following DSBs introduction, the DNA repairing process is proceeded for preventing the cell death. Generally, non-homologous end joining (NHEJ) repair generates gene disruption by insertion or deletion (indel) mutation, whereas homologous recombination (HR) repair facilitates the substitution or insertion of target sequences with the presence of donor DNA.

## 5 Genome Editing in *K. marxianus* by the CRISPR–Cas9-Mediated System

Based on encouraging experiences of *S. cerevisiae*, the CRISPR–Cas9 system has now been widely applied in various non-conventional yeast strains. Although additional improvement and optimization are required, this system exhibit tremendous promise in genome manipulation and editing in yeasts. *K. marxianus* is well recognized as a perfect host for the biosynthesis of a plethora of chemicals and bioactive compounds due to its fastest growth rate and Crabtree-negative properties (Wagner and Alper, 2016). Since NHEJ is likely to play a major role in *K. marxianus*, comparatively longer homologous arms are required to edit HR (Hong et al., 2007). A stop codon was introduced in NHEJ core genes of cell type-specific regulator (*Nej1*) and DNA ligase 4 (*Dnl4*) by altering C to T at 16 to 19 bp upstream of PAM for repressing the effect of NHEJ. This results in 100% correct HR-mediated genome editing at the *URA3* site applying a zeocin selection marker that harbor 1-kb homologous arms, leading to 4-fold improvement than that to native counterpart (Nambu-Nishida et al., 2017). Application of a wide-host range CRISPR–Cas9 system in *K. marxianus* haploid and diploid strains caused over 80% disruption of *ADE2* with 24% HR-based repair (Juergens et al., 2018). This low efficiency of HR substantiates the major role of NHEJ in DSB repair. Löbs et al. (2017b) characterized the functional genes related to the biosynthesis of ethanol and ethyl acetate in *K. marxianus* by applying the CRISPR–Cas9 system. Three different kinds of hybrid pol III promoters, such as SCR1-tRNA^Gly^, SNR52-tRNA^Gly^, and RPR1-tRNA^Gly^, were utilized to confirm the functional expression of various sgRNAs. Among the promoters, RPR1-tRNA^Gly^ presented the maximum editing efficiency (66%). Screening of alcohol-*O*-acetyltransferase (*ATF*) and alcohol dehydrogenase (*ADH*) genes corroborated that *ADH7* plays a main contribution as an alternate pathway to biosynthesize ethyl acetate. This work provides an excellent example that the CRISPR–Cas9 system has the capability of rapidly constructing gene disruption sets to characterize metabolic pathways and genes associated with the biosynthesis of valued chemicals.

**FIGURE 3:**
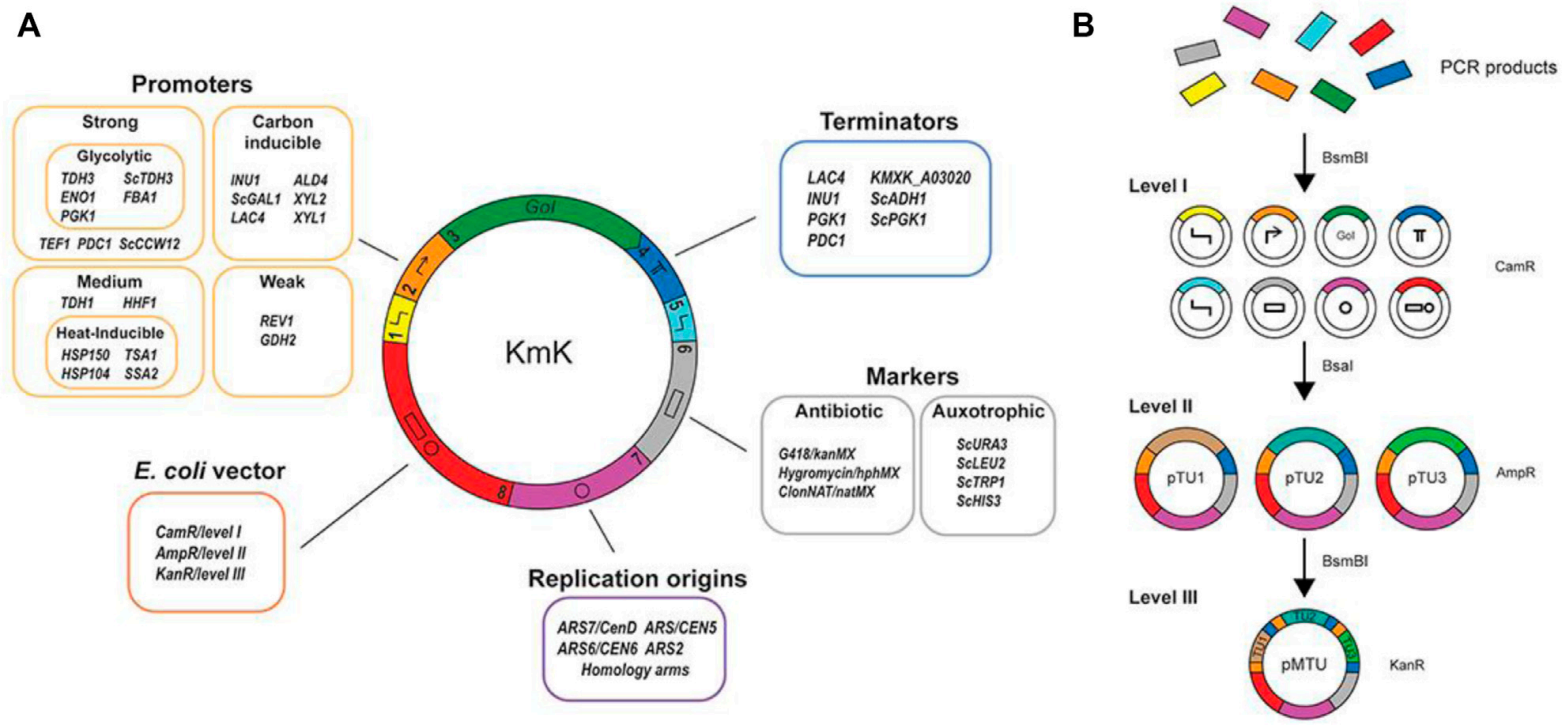
Collection of biological parts and synthetic biology tools for *Kluyveromyces marxianus*. Reproduced with permission from Rajkumar et al. (2019); an open-access article distributed under the terms of the Creative Commons Attribution License (CC BY).

The wild-type *K. marxianus* yeast is used to produce aroma and fermented products; however, the lack of standardized toolsets for metabolic and pathway engineering hinders its potential as a microbial cell factory. In this regard, Rajkumar et al. (2019) brought together and characterized an assortment of native *K. marxianus-*specific parts and vectors to express multiple genes for its genetic engineering and synthetic biology. More than 30 inducible and constitutive promoters, centromeres, terminators, and a number of known autonomously replicating sequence (ARS) elements were chosen from transcriptomic and genomic data and presented as a part of this assortment (Figure 3). All parts were cloned following the MoClo/Yeast Tool Kit standard for fast construction. They also selected various chromosomal integration sites for quick screening and developed a single-plasmid CRISPR/Cas9-based platform for genome editing and gene targeting for the rational creation of auxotrophic strains to broaden the scope of selective markers for *K. marxianus*. Overall, this collection is considered as a valued toolset for the research community interested in *K. marxianus* to build next-generation cell factories.

## 6 Concluding Remarks and Future Perspective

Thanks to recent advances in biotechnological toolsets and strategies, *K. marxianus* has become an attractive non-conventional yeast compared to *S. cerevisiae* (a model yeast) for application in food, environment, and biotechnology. This yeast is endowed with a number of exceptional attributes, such as thermal tolerance, high ethyl acetate synthesis, ability to utilize various low-cost carbon sources (i.e., xylose, lactose, and inulin), fast growth rate, and short replication time. In addition, its survival ability in the presence of high temperature and elevated concentration of ethanol indicates its enhanced adaptation capability. The tolerance capacity of *K. marxianus* has been demonstrated in numerous literature reports against stress conditions, such as acidic pH, high temperature, high ethanol concentration, and severe oxygen-depleted condition, which might be advantageous features for future industrial exploitability. Furthermore, the production of high-value fragrance and flavor biomolecules, single-cell proteins, single-cell bio-oil, and protein makes *K. marxianus* an interesting tool for food industries by expanding the product portfolio. Nevertheless, most of the investigations on *K. marxianus* have been conducted using liquid fermentation (LF) that is likely to inhibit microbial cells because of a high concentration of the final product in the fermenting medium. In order to address this setback, various approaches like medium standardization, development of new strains, cell immobilization, and removal of product were generally employed utilizing synthetic media. Due to low water content, small reactor capacity, and no additional stirring, solid-state fermentation (SSF) using waste biomasses is preferred over liquid fermentation, but longer retention time and requirement of large amounts of inoculum are the associated challenges in liquid fermentation. Thus, additional studies are needed for engineering new techniques and suitable reactor design for industrial-scale application of SSF. Currently, the exploitation of *K. marxianus* for bioethanol production using lignocellulosic biomass has gained considerable importance due to abundant accessibility of lignocelluloses. Nonetheless, the lignocellulosic depolymerization and fermentation might be hard owing to its complex architecture. In future, studies exploring various new and ecofriendly pretreatment approaches could be useful for making biomass accessibility to the microbial strains. Moreover, immobilization and evolutionary adaptation techniques are likely to enhance the efficiency of high-value products and commodity chemicals *via* economically feasible fermentative processes.

Based on literature assessment, it can be contended that the use of *K. marxianus* in industrial setting would be an exciting era. In contrast, relatively scarce information is available on the diversity of *K. marxianus* species at the physiological, metabolic, and genetic levels that necessitate novel engineering tools to biosynthesize a plethora of valuable products based on transcriptomic, genomic, and metabolic modeling data. It is also possible to reveal the mechanisms underlying the fast growth (it is believed to be fastest growing yeast on the Earth), thermoresistant, and high ability to secret proteins. It is meaningful to apprehend lack of knowledge around genes, metabolic pathways, key enzymes, and their regulation for a complete insight into mechanism for producing relevant metabolites by *K. marxianus*. Exploitation of coordination between functional diversities and genetic variation at the level of species might be a potential research prospect for advancing this field. In conclusion, it can be envisioned that *K. marxianus* is useful in a large number of biotechnological applications, but an in-depth acquaintance with the genetic diversity remains to be elucidated. In this review, we outline the diverse applications of *K. marxianus* in different industrial sectors that would pave the way for exploring new opportunities in the food, feed, biotechnological, environmental, and pharmaceutical industries.
